# Clinical presentation, diagnosis and management of aerodigestive tract foreign bodies in the paediatric population: Part 2

**DOI:** 10.4102/sajr.v25i1.2027

**Published:** 2021-03-23

**Authors:** Rishi P. Mathew, Teresa I-Han Liang, Ahamed Kabeer, Vimal Patel, Gavin Low

**Affiliations:** 1 Department of Radiology, Rajagiri Hospital, Aluva, India; 2 Department of Radiology and Diagnostic Imaging, Faculty of Medicine and Dentistry, University of Alberta Hospital, Edmonton, Canada; 3 Department of Pediatric Surgery, Rajagiri Hospital, Aluva, India

**Keywords:** bronchoscopy, button battery, endoscopy, foreign bodies, ingestion, magnet

## Abstract

Children, especially toddlers, because of their behaviour, physiology and anatomical characteristics such as oral exploration of their surroundings, have a tendency to place objects in their mouth. Therefore, ingestion or aspiration of foreign bodies (FBs) in children is a potentially life-threatening and common problem seen across the world. In this second part of our pictorial review on ingested and aspirated FBs, we focus on the paediatric population, reviewing the current literature and examining the epidemiology, clinical presentation, anatomic considerations, appropriate imaging modalities, key imaging characteristics associated with clinically relevant FBs in the emergency department (ED) and current management protocols.

## Introduction

Aerodigestive foreign bodies (FBs) in the paediatric population are potentially life threatening and are frequently encountered in hospital emergency rooms (ERs). Identification of these FBs in the aerodigestive tract can be challenging because of non-specific symptoms, lack of a clear history or a combination of both. Imaging plays a crucial role in the diagnosis of ingested and aspirated FBs in children and can be vital in guiding the clinical management of these patients. This article reviews the current literature for common presentations of both FB ingestion and aspiration, as well as the current recommendations for their appropriate evaluation and management.

## Discussion

### Foreign body ingestion

Children aged 5 years and below account for approximately 70 000 cases of FB ingestion annually in the United States alone, with a peak incidence reported between 6 months and 3 years.^1^ In most cases (80% – 90%), although the FB will pass through the gastrointestinal tract (GIT) without requiring intervention, 10% – 20% will require endoscopically assisted FB retrieval and 1% will require surgical intervention for the extraction of a FB or to treat a complication.^2,3^

Clinical symptoms vary with the age of the patient, size and location of the FB. Symptoms such as drooling, gagging and poor feeding are common presentations in infants affected with ingested FBs, whilst older children may present with odynophagia, dysphagia and chest pain. When the FB is in the proximal or mid-oesophagus and close to the airways, children may present with cough, wheezing or symptoms of respiratory distress. In the absence of mucosal injury or obstruction, FBs in the stomach or bowel are less likely to cause symptoms; when symptoms present, these include abdominal pain, vomiting and hematemesis. In most cases, retained FBs in the paediatric age group are not dramatic.^4^ The most commonly ingested FBs in descending order are as follows: coins, magnets, batteries, small toys, jewellery, buttons and bones.^5^

#### Coins

**FIGURE 1:**
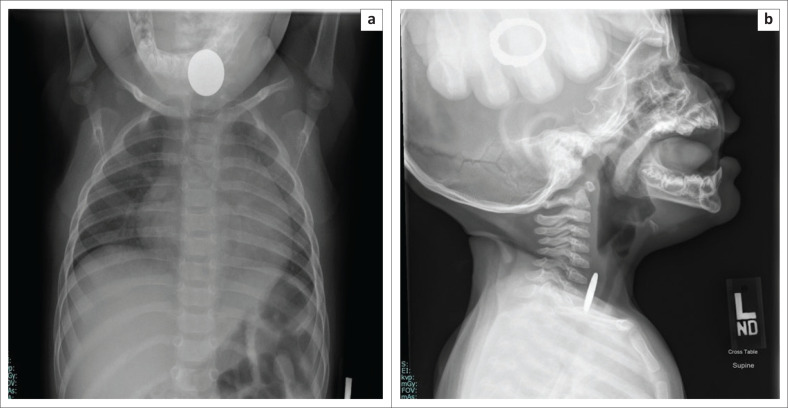
Frontal (a) and lateral (b) radiographs of the neck reveal a swallowed coin in the proximal oesophagus of a 10-month-old child who presented with respiratory distress and drooling.

**FIGURE 2:**
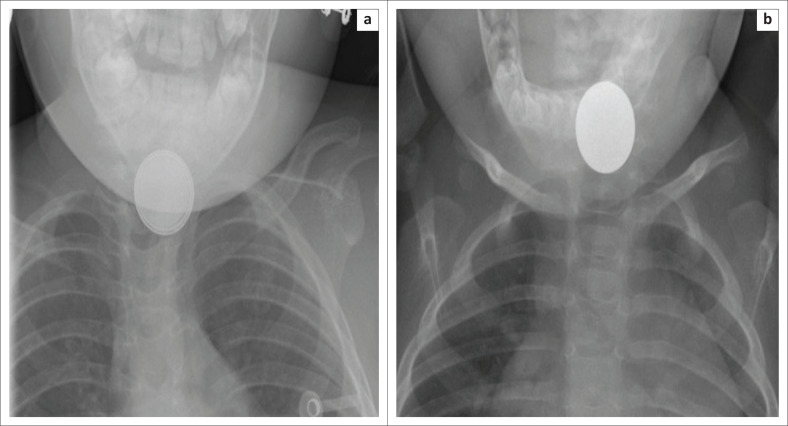
Magnified frontal radiographic images of the neck of two different patients with an ingested button battery (a) and a coin (b). A button battery has a ‘halo sign’ or a double density at its periphery en face, whilst a coin appears as a discoid radiodense object of uniform density.

Coins are the most commonly ingested FBs in children. The initial evaluation of a child suspected of coin ingestion is radiography of the neck, chest and abdomen. Apart from regular anterior–posterior (AP) views, a lateral view of the neck can be obtained to better localise the coin. A coin is recognised on a radiograph by its metallic density and flat disk shape. On AP and lateral views, a coin in the oesophagus will appear as a radiodense circular object (‘en face’) and as a thick line (‘on edge or in profile’), respectively (Figure 1a and b). In comparison, a coin in the trachea will appear as a dense circular object on the lateral view and as a thick line on the AP view, as the cartilage prevents the alternate appearance that would be expected with an FB in the oesophagus. It is of utmost importance for the radiologist to differentiate between an ingested coin and a button battery (BB), as the latter requires emergency removal. A BB lodged in the oesophagus can be immediately recognised as it has a ‘halo sign’ or a double density at its periphery en face, whilst a coin appears as a discoid radiodense object of uniform density (Figure 2a and b).^6^

As coins lack sharp edges and are generally non-toxic, in most cases they pass into the stomach and through the GIT without any complications. Nearly 75% of coins in the distal oesophagus pass spontaneously into the stomach within the first 6–10 h following ingestion. The spontaneous passage rates for coins in the mid- and proximal oesophagus into the stomach are reportedly estimated to be 43% and 14%, respectively.^6^ Coins with a diameter of > 2.3 cm (e.g. an American quarter) are at risk for oesophageal retention.^7^

**FIGURE 3:**
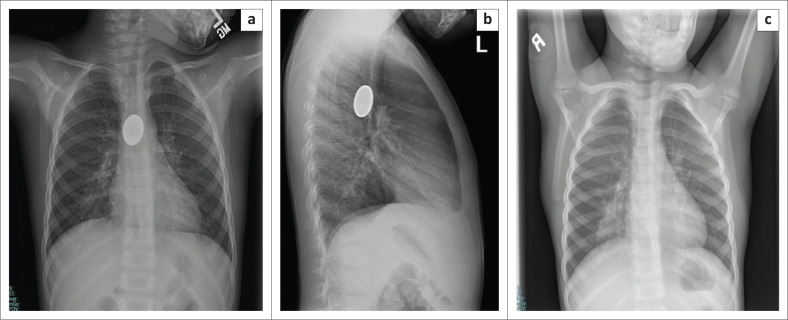
Initial frontal (a) and lateral (b) chest radiographs demonstrate a coin in the mid oesophagus of a 4-year-old male child. A follow-up radiograph (c) indicates that the coin has passed into the stomach. The child was managed conservatively.

**FIGURE 4:**
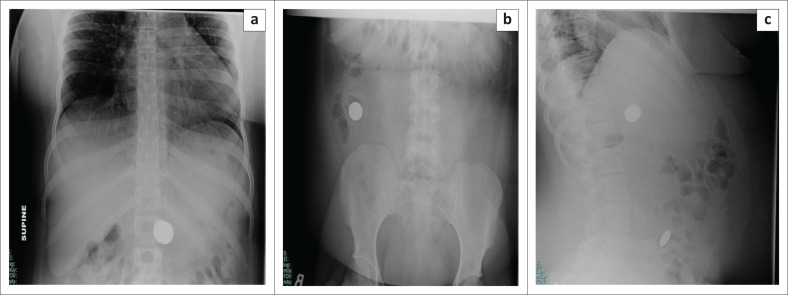
Serial abdominal radiographs frontal (a and b) and lateral (c) views of a 16-year-old female patient with a psychiatric history who presented with multiple coin ingestions and was managed conservatively.

Asymptomatic children can be managed conservatively once the coin has reached the stomach with anticipatory advice provided to the parents or caregivers to check the stool for confirmation of coin passage (Figures 3a, b and 4a–c).^6,7^ If passage of the coin has not been observed in the stool, a radiograph of the abdomen should be performed, and if it is retained in the stomach 4 weeks post ingestion, the coin should be removed endoscopically.^6^

#### Magnets

**FIGURE 5:**
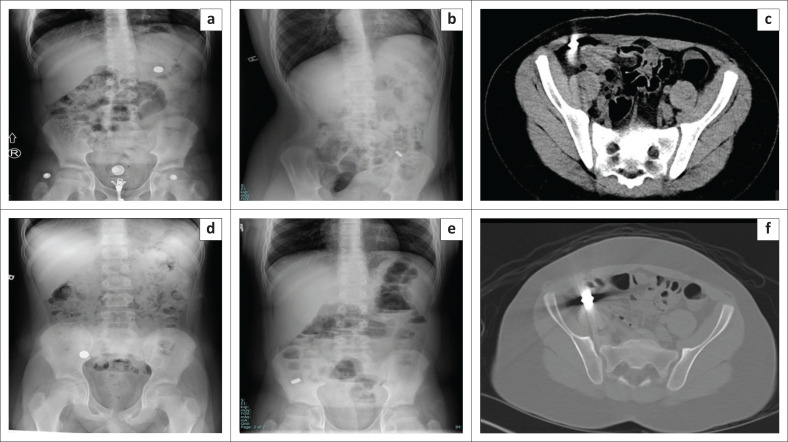
Serial abdominal radiographs and computed tomography (CT) images of a 12-year-old asymptomatic male child who swallowed five small magnets, shows the magnets initially in (a) the stomach, (b) then in the left lower quadrant and (c, d and e) later in the right lower quadrant. The multiplicity of magnets can be better appreciated on CT (c and f). The magnets were subsequently removed by colonoscopy.

Given the extensive availability of magnets in toys, ingestion of paediatric magnetic FB remains a serious and increasing public health hazard. When multiple magnets are ingested, they can become attached to each other in-between intestinal bowel loops. As these are unlikely to become disengaged, the resultant pressure can cause necrosis within a couple of hours leading to complications, such as bowel obstruction or perforation, fistula formation, volvulus, peritonitis and sepsis.^6,8^ The risks are especially high when more than one magnet has been ingested. Identification of one or more magnets can be challenging on a single radiograph, and it is recommended that at least two views (frontal and lateral) should be acquired (Figure 5a–f). The identification of a gap between the magnets should raise concerns for bowel entrapment and the risk of ischaemic injury, necessitating the need for an urgent surgical intervention.^8^

Indications and the timing for intervention are dependent on several factors, such as, patient age, anatomic location, symptoms and time since ingestion. In the case of a single-magnet ingestion, confirmed by radiography and without any complications, an endoscopic removal may not be necessary, but should be considered when radiography cannot confirm the number of the magnets ingested, the patient is at risk for further ingestion, there is a lack of means for close follow-up or the patient demonstrates clinical features of obstruction (e.g. pain, vomiting, tachycardia, etc.). In centres lacking an endoscopy service, the caregivers should be instructed to assess the progress and confirm passage of the magnet by obtaining serial radiographs of the abdomen along with stool surveillance. Once a solitary magnet has traversed the oesophagus, spontaneous passage through the GIT is highly likely. Stool softeners or polyethylene glycol can mitigate a delayed transit. Urgent endoscopy or surgery should be considered in those in whom the magnet has remained stationary.^9,10^

In children with multiple ingested magnets, even if asymptomatic, current guidelines stipulate their urgent removal when the location is approachable endoscopically, either by oesophagogastroduodenoscopy or by colonoscopy. The type of retrieval device used varies depending on the size and shape of the ingested magnet, although the preferred instrument for small, round magnets is a retrieval net (Roth Net, US Endoscopy). No clear consensus exists for the management of multiple magnets in asymptomatic children, which have crossed the duodenal–jejunal flexure but remain proximal to the small bowel. The options for management in such cases include endoscopic removal by small bowel enteroscopy (single or double balloon), removal by laparotomy or laparoscopy with concurrent increased morbidity, mortality and costs, or conservative management.^10^

A 2012 survey involving 424 children with magnetic FB ingestion over a 10-year period revealed that 52% of the patients were managed by endoscopy alone, 20% required both endoscopic and surgical intervention, 8% were managed surgically and 15% were managed conservatively. Perforation or fistula repair was required in 41% of the surgically managed cases and 22% necessitated partial bowel resection.^10^

#### Button or disk batteries

**FIGURE 6:**
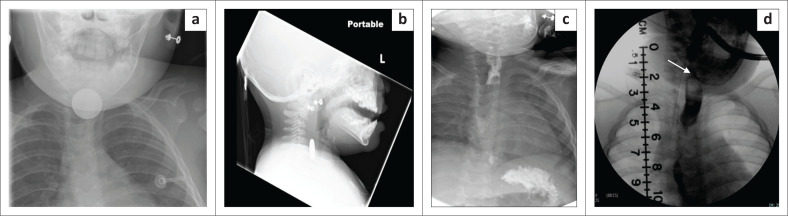
Frontal (a) and lateral (b) radiographs of the neck reveal a button battery in the cervical oesophagus. An initial radiograph (c) carried out 3 days post ingestion shows mild irregularity at the site of the button battery impaction, but no contrast extravasation was observed. Follow-up fluoroscopy (d) performed 3 months later demonstrates a stricture (arrow) involving the cricopharyngeal portion.

Button batteries are now commonly used in many items, such as watches, key fobs, toys and remote controls. Of all the sites, impaction of a BB at the oesophagus poses the highest risk, and as a result, oesophageal BBs are the most critical indication for emergency endoscopy in the paediatric population,^10^ with the risk of clinically significant oesophageal injury within 1–2 h.^11^ In addition to the low-voltage currents and pressure necrosis, the leaking alkaline solution from the BB has a direct corrosive effect, capable of causing rapid liquefactive necrosis, leading to oesophageal mucosal injury as early as 1 h post-ingestion, forming the main mechanism of injury in BB ingestion.^12^ Oesophageal perforation can occur as early as 6 h, with other complications including oesophageal stricture (Figure 6a–d), scarring, tracheo-oesophageal fistula or oesophageal–aortic fistula with life-threatening haemorrhage.^13^

Studies have shown that the outcome of BB ingestion is mainly dependent on the battery size. Minor and moderate complications were more commonly reported in children who swallowed batteries with diameters > 15 mm, and major complications were seen in those who swallowed batteries > 20 mm in diameter.^13^ Because of the inherent danger associated with it, the radiologist needs to immediately distinguish radiographically a BB from a coin, a common mimicker. When evaluating a child suspected of BB ingestion, it is important to obtain both frontal and lateral radiographs from the nasopharynx to the anus, as the two views can help to differentiate a BB from a coin.^14^ As mentioned earlier, BBs have a distinctive appearance on radiographs. On frontal radiographs, BBs have a halo or double-density shadow caused by the bilaminar structure of the battery. Additionally, on the lateral view, a BB may show the step-off at the junction of the cathode and anode, whereas a coin on AP and lateral radiograph appears as a radiodense circular object and a thick line, respectively.^4,6,13,14^

**FIGURE 7:**
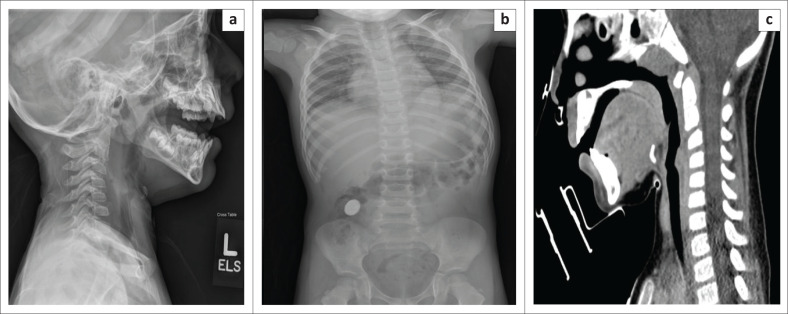
Lateral radiograph of the neck (a) and abdominal radiograph (b) of a 3-year-old child taken 3 days after ingestion of a button battery shows mild thickening of the retropharyngeal soft tissue with the foreign body located in the distal ileum. Computed tomography (c) revealed that the retropharyngeal soft tissue noted on the neck radiograph was actually collapsed oesophagus with no collection. The child was managed conservatively without any complications.

Once the battery has passed into the stomach or beyond, the need for endoscopic intervention is dependent on multiple factors, such as the age of the child, presence of symptoms and time since the ingestion. Endoscopic intervention is required for BBs > 15 mm – 20 mm in diameter, children < 5 years of age, presence of symptoms and longer time after ingestion because of the increased risk of morbidity. Asymptomatic older children with ingested BBs < 20 mm in size may be managed conservatively (Figure 7a–c) by outpatient observation with repeated radiographs every 3 – 4 days.^6,13,15^

In contrast to BBs, ingestion of cylindrical batteries is less commonly seen in children. In a study involving 8648 cases of battery ingestion over an 18-year period, more than 94% of the ingested cells were BBs, whilst less than 6% of cases included cylindrical battery ingestion.^16^ Cylindrical batteries are infrequently associated with minor or moderate symptoms, and major life-threatening complications are rare. Batteries retained in the stomach > 48 h should be removed endoscopically. Asymptomatic children with cylindrical batteries located beyond the stomach may be managed conservatively by checking the stool for its passage and with follow-up radiographs over 10 – 14 days.^17^

#### Glass and sharp objects

Sharp objects account for approximately 5% – 30% of all swallowed objects, such as pins, needles and paper clips.^18^ Children may also swallow glass pieces or its products, such as beads and marbles. Most sharp objects, including glass, are radio-opaque and will be visible on a radiograph, all though the size and location of the glass can affect its visibility.

**FIGURE 8:**
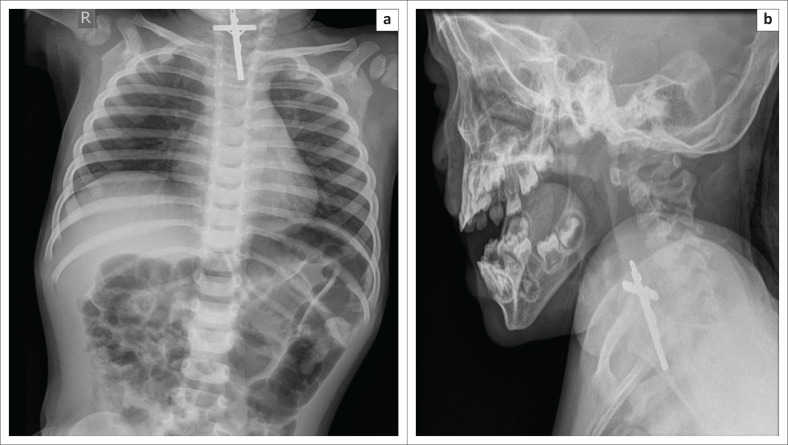
Frontal (a) and lateral (b) radiographs of the neck indicates a crucifix impacted in the cervical oesophagus of a 2-year-old child. The foreign body was retrieved by rigid oesophagoscopy.

**FIGURE 9:**
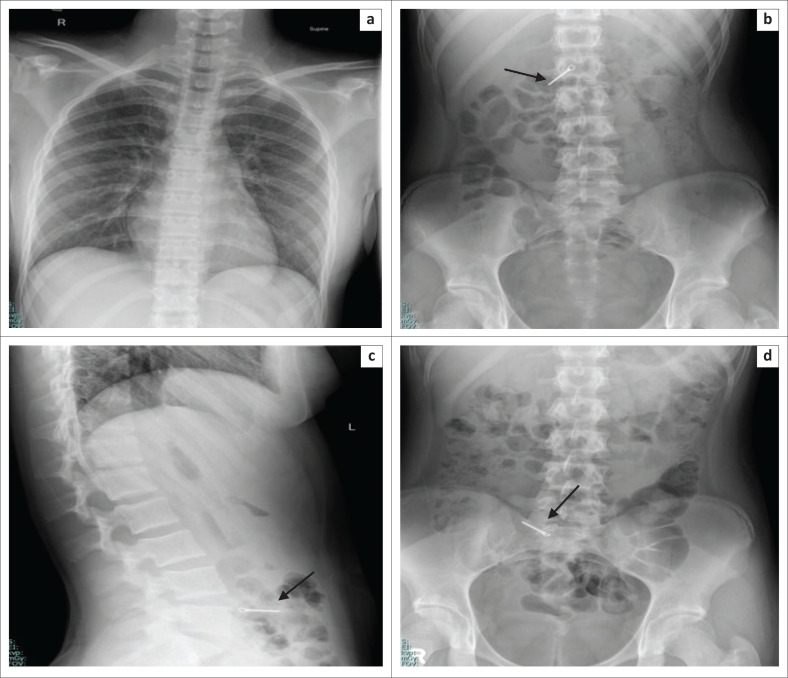
Serial radiographs of a 14-year-old-female patient who accidentally swallowed a loose metallic fragment (arrow) from her dental braces. The foreign body had been transmitted from the oesophagus (a) through the gastric pylorus (b), and later into the right lower quadrant (c and d) on follow-up serial radiographs. The patient was managed conservatively without any complications.

Because of the risk of oesophageal perforation, sharp objects located proximal to the gastric pylorus are removed endoscopically (Figure 8a and b).^18^ Even if a sharp object has passed into the small bowel, it needs to be followed daily with radiographs until it passes through the GIT (Figure 9a–d) because of the 35% risk of complications.^18^ Surgery is recommended if the sharp object fails to move through the bowel after 72 h, indicating impaction. Although perforation can occur anywhere, the most common site is the ileo-caecal region, especially at the appendix or a Meckel’s diverticulum. In the absence of symptoms, blunt glass objects such as marbles and beads may be managed conservatively. However, glass pieces with sharp margins located in the oesophagus or stomach should be removed endoscopically.^18^

#### Radiolucent foreign bodies

**FIGURE 10:**
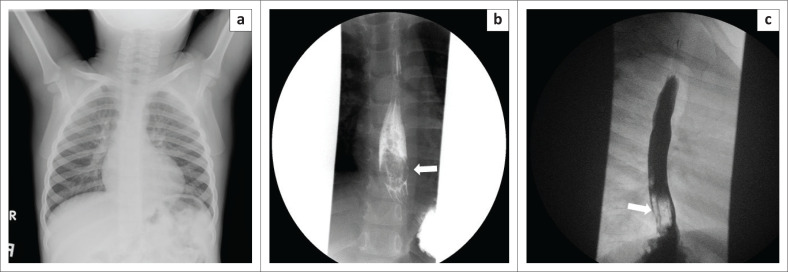
Images of a 3-year-old child who ingested a toy part (toy car tyre). The chest radiograph (a) was unremarkable. Upper oesophagogram, frontal (b) and oblique (c) views, showed a ring-shaped filling defect in the lower oesophagus, immediately proximal to the gastro-oesophageal junction with contrast traversing into the stomach without any delay.

At least ≥ 35% of the FBs ingested are radiolucent, and these include plastic objects (e.g. majority of the toys) (Figure 10a–c), some fish bones and plant materials (e.g. wort, thorns, splinters, etc.). In these cases, radiographs will be unhelpful, and management is guided mainly by clinical presentation. In such situations, imaging with fluoroscopy or CT may prove useful, especially in patients with suspected complications. Asymptomatic patients can be managed conservatively.^18^

A summary of the various indications for a conservative approach, endoscopy or surgery in the management of ingested FBs in the paediatric population as per literature review has been shown in Table 1.^10,15,19^

**TABLE 1:** Indications for ingested foreign body management in the paediatric population by conservative approach, endoscopy or surgery according to literature review.

Object type	Conservative management	Endoscopic removal	Surgery
Coins	A coin in the stomach of an asymptomatic patient can be managed conservatively, with anticipatory guidance given to caregivers to check all stools for coin passage.^10^	Symptomatic patients, coins remaining longer than 12 – 24 h in the oesophagus and 3 – 4 weeks in the stomach even in an asymptomatic patient warrant endoscopic removal.^19^	-
Magnets	Magnets beyond endoscopic reach and showing movement on serial radiographs.^15,19^	Magnets within endoscopic reach is a reason for urgent endoscopy.^10,15^	Failure of a magnet to move through the lumen on sequential radiographs, and location beyond endoscopic reach, should prompt surgical evaluation; radiographic findings suggesting bowel entrapment (detection of a gap between magnets on imaging), obstruction or perforation should prompt emergent surgical evaluation.^19^
Button Batteries	In older asymptomatic children with gastric button batteries < 20 mm in size, one can consider outpatient observation with a repeat radiograph within 48 h.^15^	Emergent endoscopic removal is indicated for a suspected disk battery discovered in the oesophagus and those remaining in the stomach for > 48 h.^10,15^	Formal laparotomy with removal should be considered if it appears that the passage of the battery in the bowel has been arrested.^10,15^
Sharp and pointed objects	Sharp objects passing the duodenum should be followed radiographically daily to document passage. Such cases should be managed cautiously, because 15% – 35% of sharp objects that pass the stomach cause intestinal perforation, usually in the area of the ileocecal valve.^19^	Sharp objects located in the oesophagus, stomach or duodenum require urgent endoscopic removal; endoscopy should still follow a radiological examination with negative findings because many sharp-pointed objects are not radiographically visible.^10,19^	If the sharp foreign body beyond the duodenum fails to progress radiographically for three consecutive days, surgical intervention should be considered, as well as in patients with failed endoscopic attempts.^19^
Long and short blunt objects	Objects with a diameter < 2.5 cm and < 6.0 cm in length may be managed conservatively by observing serial radiographs for passage.^15^	Objects having a diameter > 2.5 cm and longer than 6 cm in length are unlikely to pass the pylorus and the duodenal sweep, respectively, and hence, require endoscopic removal.^15^	Surgical removal should be considered if objects remain in the same location distal to the duodenum for more than 1 week.^19^
Bezoars	-	In the acute clinical setting, endoscopic disruption and removal of the mass can be performed.^19^	Many bezoars require surgical removal.^19^

*Source*: Kramer et al.^10^; Gurevich et al.^15^ and Guelfguat et al.^19^

Note: Please see the full reference list of the article, Mathew RP, Liang TI-H, Kabeer A, Patel V, Low G. Clinical presentation, diagnosis and management of aerodigestive tract foreign bodies in the paediatric population: Part 2. S Afr J Rad. 2021;25(1), a2027. https://doi.org/10.4102/sajr.v25i1.2027, for more information.

### Foreign body aspiration

Foreign body aspiration (FBA) is a serious condition commonly reported in childhood, which requires urgent intervention to prevent complications and irreversible lung injuries. Foreign body aspiration globally remains the fourth most common cause of accidental deaths amongst infants and preschool children,^20^ and the third most common cause of death amongst infants in the United States.^21^ Children under the age of 3 years are most commonly affected. The enhanced risk for this age group is attributed to poorly developed posterior dentition, underdeveloped mechanism of deglutition and airway protection, and the inherent tendency of this age group to place objects into their mouth.^22^

**FIGURE 11:**
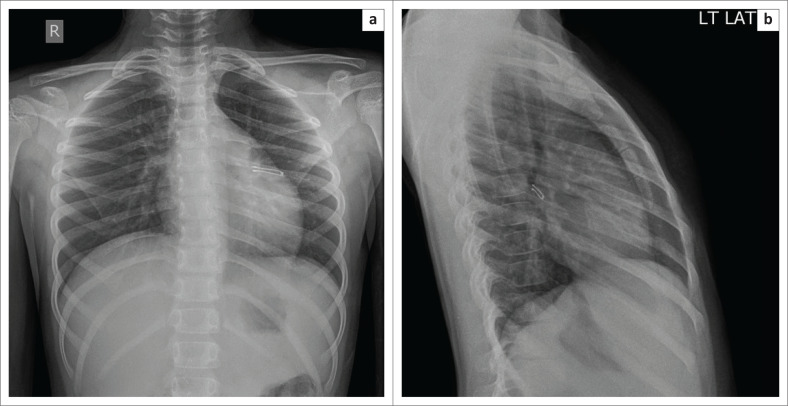
Frontal (a) and lateral (b) radiographs in a 3-year-old child who aspirated a light emitting diode bulb into the left main stem bronchus.

The clinical presentation and complications associated with FBA depend on the location of the FB, extent of airway obstruction, age of the child, FB type and the time elapsed following aspiration. Hence, the classic triad of cough, wheeze and reduced breath sounds is not always present. Most aspirated FBs are organic, primarily nuts (40%, such as peanuts) and seeds, with the remaining being non-organic materials such as coins, toys and balloons. However, the type of FBs aspirated varies amongst countries, and are largely dependent on cultural and socio-economic factors, as well as parental influence, eating habits and patient age.^23^ For example, sunflower, pumpkin and watermelon seeds are the most commonly aspirated FBs in the paediatric population from countries, such as Egypt, Turkey and Greece, whilst bones are more commonly reported in patients from China and South Asian countries.^24^ Recently, an increasing incidence of ‘scarf pin-related Hijab syndrome’ has been reported in the literature amongst female adolescent patients from countries with a large Muslim population who wear headscarves and place the safety pin in their mouth prior to securing the veils. Accidental aspiration of the pins occurs with talking, laughing or coughing.^25,26^ Unlike in adults in whom the majority of the aspirated FBs lodge in the right main bronchus, the left main bronchus is equally affected if not more involved in children, especially in those aged ≤ 3 years (Figure 11a and b).^26^ The type of FB aspirated also plays a decisive role in the site of lodgement. For example, scarf pins more often lodge in the left bronchial tree. Many have attributed this to the Bernoulli phenomenon, which states that coughing, laughing or talking creates a strong negative pressure within the narrow-left bronchus in comparison with the much wider right bronchus.^26^

Diagnosis of FBA may be complicated by a delay in presentation or from an inaccurate diagnosis in asymptomatic children or in those presenting with non-specific symptoms. Delayed diagnosis or retained FBs in the airways can result in chronic cough or wheezing, or lead to complications ranging from recurrent pneumonia, bronchiectasis, asthma, lung collapse and lung abscess to potentially fatal airway obstruction.^27,28^ In older children, the most commonly presenting symptom is cough, whilst wheezing and stridor, excessive crying, seizures and loss of consciousness are more commonly reported in infants. Episodes of choking or coughing spells have a reported sensitivity of 80% – 82% and a specificity of 34% for FBA.^29^

**FIGURE 12:**
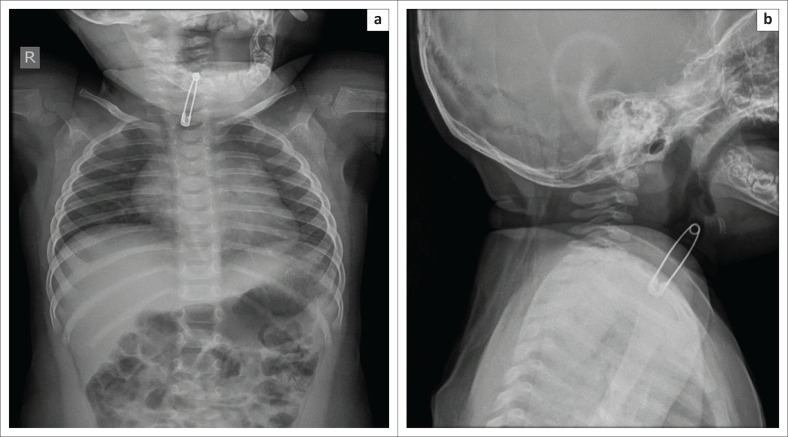
Frontal (a) and lateral (b) radiographs of the neck in a 2-year-old child with an aspirated safety pin in the subglottic region.

Currently, the three main imaging modalities used for evaluating paediatric patients with FBA include radiography (Figure 12a and b), fluoroscopy and multi-detector computed tomography (MDCT).^30^ As the majority of the aspirated FBs are organic, with the most common being a peanut, these tend to be radiolucent and unidentifiable on a radiograph.^31^ Plain radiography is the first-line imaging modality for evaluating patients with FBA, and the reported sensitivities and specificities range from 66% – 88% and 30.0% – 71.4%, respectively.

**FIGURE 13:**
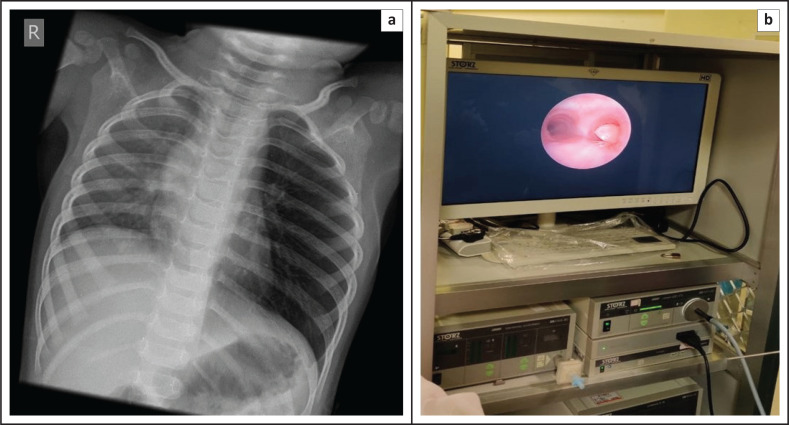
Chest radiograph (a) of a 2-year-old child who aspirated a peanut into the left main bronchus with resultant air trapping and hyperinflation of the left lung. Bronchoscopy (b) confirmed the aspirated peanut in the left main bronchus.

**FIGURE 14:**
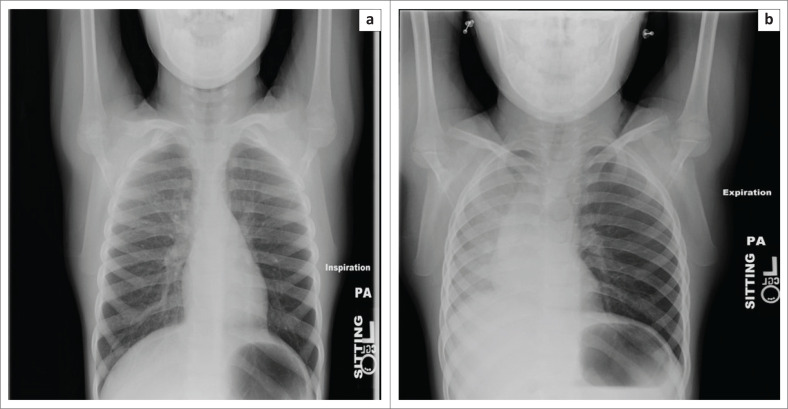
Images of a 6-year-old child who aspirated a candy wrapper into the left upper and lower segments of the left bronchus whilst eating a chocolate. The left lung appears to be hyperlucent on inspiration (a) and shows air trapping with mediastinal shift to the right on expiration (b).

The most common findings include air trapping or hyperinflation (35%) and atelectasis (16%).^31^ A normal chest radiograph does not exclude FBA, and a high index of suspicion is required especially in patients with a suggestive history, witnessed choking episodes by a caregiver or clinical signs on examination. Both inspiratory and expiratory chest radiographs should be requested when evaluating for FBA; conventional chest radiographs are taken in full inspiration. In the absence of FBA, both lungs will appear well inflated and uniformly radiolucent on an inspiratory film, whilst on the expiratory film, the lung volumes will be reduced with symmetric mildly increased radiodensity. An airway FB can partially obstruct the airway by creating a ‘ball-valve’ mechanism, allowing air to enter the lung as the bronchus dilates during inspiration permitting sufficient patency of the lumen at the site of obstruction, whilst during expiration, the lumen narrows, occluding the affected bronchus resulting in obstructive emphysema, sometimes referred to as ‘air trapping’ (Figures 13a and b and 14a and b).^32,33^

**FIGURE 15:**
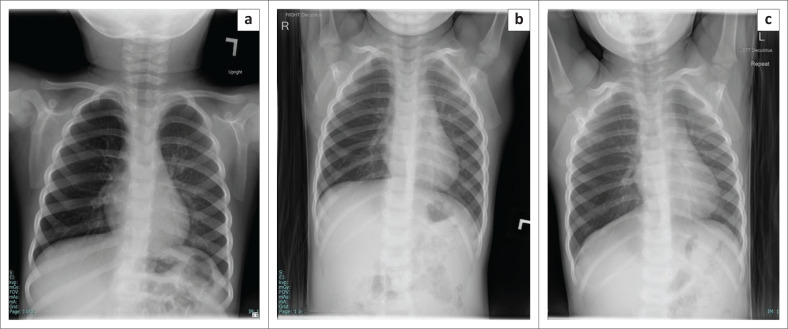
Images of a 30-month-old child with a history of almond aspiration. Although the foreign body was not radiographically visible, the chest radiograph (a) showed a hyperinflated right lung, and right lateral decubitus inspiratory (b) and expiratory films (c) demonstrated air trapping, suggesting foreign body aspiration into the right main bronchus.

Obtaining satisfactory chest radiographs during the desired phases of respiration may not be possible in some younger children, especially in infants from a lack of cooperation. Such situations may be overcome by demonstrating air trapping on the lateral decubitus film. When a child is placed on his or her side, the dependent hemithorax gets splinted, preventing movement of the dependent lung, thereby causing it to be under aerated, as well as resulting in narrowing of the intercostal spaces and elevation of the hemidiaphragm, whilst the contralateral or anti-dependent lung remains well aerated. If air trapping is present, the affected lung, lobe or segment will remain hyperlucent when that side of the thorax is in the dependent position (Figure 15a–c).^33^ However, one must keep in mind that air trapping is not specific for FBA, as it can also be seen with any lesion partially obstructing the airways such as asthma with mucus plugging or pulmonary infections, both of which can simulate FBA.^34^ As a result, some experts argue the clinical benefit of expiratory films. A recent cohort involving 328 paediatric patients with suspected FB aspiration found that the addition of expiratory views increased the false positives with the test accuracy remaining low, thereby questioning the benefit of the additional technique.^35^ Radiographic findings apart from air trapping that may be seen in patients with FBA include identification of a radio-opaque FB, consolidation, atelectasis, bilateral hyperinflation, pleural effusion, pneumothorax and bronchiectasis. About 24% – 30% of the patients with FBA may have a normal chest radiograph.^30,34,35^

In the past, fluoroscopy was used in paediatric patients with suspected FBA to document mediastinal shift or reduced movement of the diaphragm from air trapping, especially in patients with inconclusive or normal chest radiographs or non-cooperative patients. This operator-dependent modality now has only a limited role because of the wider availability of MDCT, although fluoroscopy may still prove useful in centres where MDCT is unavailable. During the examination, the child is placed in a supine position on the fluoroscopy table with the upper and lower limbs stabilised by an assistant. The field of view is adjusted to cover both thoraces appropriately, whilst the chest is carefully examined in real time during various phases of breathing to detect abnormal side-to-side mediastinal shift or reduced diaphragmatic movement.^35^ Fluoroscopy is normal in 53.0% of the patients with FBA, and the reported sensitivities and specificities of fluoroscopy for FBA are 46.9% – 80.0% and 55.0% – 94.6%, respectively.^36^

**FIGURE 16:**
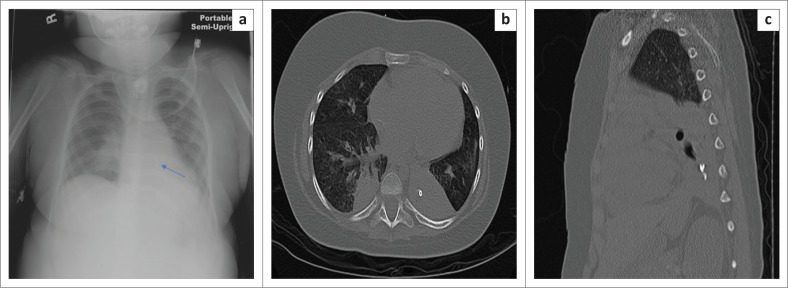
Images of a 6-year-old child with severe anoxic encephalopathy who presented with a history of teeth aspiration. The aspirated teeth are barely visible (arrow) on the chest radiograph (a). Multi-detector computed tomography axial (b) and sagittal reformatted (c) images confirmed the location of the aspirated teeth in the left lower lobe bronchus.

Multi-detector computed tomography is the most sensitive imaging modality for diagnosing FBA; however, because of increased radiation exposure, it is generally reserved for elusive cases. Unlike radiography, the advantage of MDCT is that it can demonstrate and precisely locate both radiolucent and radio-opaque FBs in the tracheo-bronchial tree prior to bronchoscopy, as well as identify subtle air trapping (Figure 16a and b). An additional advantage of MDCT is that it can be used for the evaluation of patients with suspected residual FB after bronchoscopy removal, which is thought to occur 1% – 18% of cases.^37^

#### Nasal and posterior nasopharyngeal foreign bodies

Foreign bodies in the nasal cavity make up approximately 0.1% of paediatric presentations to the ER.^38^ Children place FBs in their nasal orifices for various reasons, such as curiosity, ease of availability and intellectual disability (in older children).^38^ The majority of the children who present with nasal FB insertion are between 3 and 4 years of age, and these FBs come in various sizes and shapes. Approximately 23% – 46% of all paediatric nasal FBs are toys and 12% – 27% are organic (food-related).^38^ The right nasal cavity (60%) is more commonly affected than the left (34.9%), mostly attributed to the predominance of right-handedness.^39^ The majority of the FBs are immediately removed in the ER or out-patient clinic itself, as anterior rhinoscopy allows direct visualisation to localise and identify the FB.^39^ Paediatric patients with nasal FBs are referred to ENT specialists when the objects are not visualised in the ER following anterior rhinoscopy. Of all the FBs, BBs can pose a serious challenge requiring general anaesthesia and specialised instruments for extraction. Complications associated with BB insertion into the nose include nasal cavity adhesion, septal perforation and saddle nose. Diagnostic imaging is generally reserved for those cases where nasal FBs are clinically suspected but careful physical examination and nasal endoscopy failed to reveal the same.^39^

**FIGURE 17:**
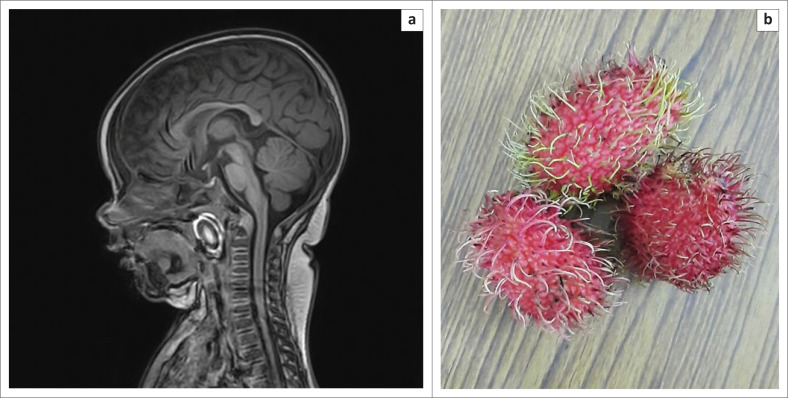
Sagittal reformatted magnetic resonance imaging brain image (a) shows the first documented case of nasopharyngeal lodgement of an aspirated rambutan fruit (b) in a 6-month-old child who was brought to the emergency room with asphyxiation and cardiac arrest. Magnetic resonance imaging brain was performed to evaluate for encephalopathy-related changes when the exotic fruit was identified in the nasopharynx.

The posterior nasopharynx is a rare site for FB lodgement and hardly receives mention even in standard textbooks. Most of the documented literature available on posterior nasopharyngeal FBs are case reports, and reported objects include a ring, a tooth, a leech and even a fish.^40^ The transit and subsequent impaction of a FB into the posterior nasopharynx can occur from various cases, such as forceful emesis and coughing causing an upward migration of the FB from the pharynx or oesophagus into the posterior nasopharynx, migration of a FB into the posterior nasopharynx during extraction attempts, traumatic penetration into the posterior nasopharyngeal space or an iatrogenically placed posterior nasopharyngeal FB after surgery. The diagnosis of a posterior nasopharyngeal FB can be challenging as the symptoms are variable and some patients may remain asymptomatic.^40,41^ Radiologists need to be aware of the posterior nasopharynx as a possible site for FB impaction, especially in patients with a presenting history of aspiration, as in rare situations, FBs may be discovered in the posterior nasopharynx on imaging studies performed for evaluation of other regions of the body (Figure 17a and b).

Rigid bronchoscopy is considered the gold standard for both the diagnosis and management of FBA in the paediatric population, especially for FBs lodged within the trachea. However, many consider optimal two-step approach, whereby the diagnosis of FBA is confirmed using flexible bronchoscopy followed by a therapeutic rigid bronchoscopy, whilst some favour flexible bronchoscopy for both diagnosis and management. Both techniques have advantages and disadvantages, as elaborated in Table 2.^42^ The incidence of complications associated with bronchoscopy ranges from 2.9% to 9% with a reported mortality rate of 0% – 1.5%. The reported complications include hypoxemia, tracheal or bronchial laceration or haemorrhage, laryngeal oedema, broncholaryngospasm, pneumothorax, pneumomediastinum, re-intubation, mechanical ventilation, pneumonia, cardiac arrest and anoxic brain injury.^43^

**TABLE 2:** Rigid bronchoscopy versus flexible bronchoscopy, the advantages and disadvantages.

Variable	Rigid bronchoscopy	Flexible bronchoscopy
Advantages	Allows safe ventilation and the ease of use of both telescopic lens and grasping forceps during the extraction of sharp or large foreign bodies. Allows a wider operative view. Allows optimal aspiration in cases of massive haemorrhage. Provides the added function of an endotracheal tube and its ability to secure the airway, especially in children with asphyxiating foreign bodies.	Comparatively easier and safer procedure in experienced hands. Can be performed under local anaesthesia. Comparatively inexpensive. Because of its flexible nature, it is useful in distal foreign bodies, mechanically ventilated patients, patients with spine injuries, jaw or skull fractures that may be complicated by excessive traction required for rigid bronchoscopy.
Disadvantages	Requires prolonged general anaesthesia. Requires the skills of an experienced endoscopist. More invasive. Limitations of use when the foreign bodies are lodged in the peripheral airways.	The main drawback is the suboptimal control of the main airways in the case of haemorrhage.

*Source*: De Palma A, Brascia D, Fiorella A, et al. Endoscopic removal of tracheobronchial foreign bodies: Results on a series of 51 pediatric patients. Pediatr Surg Int. 2020;36(8):941–951. https://doi.org/10.1007/s00383-020-04685-1

### Medical devices

**FIGURE 18:**
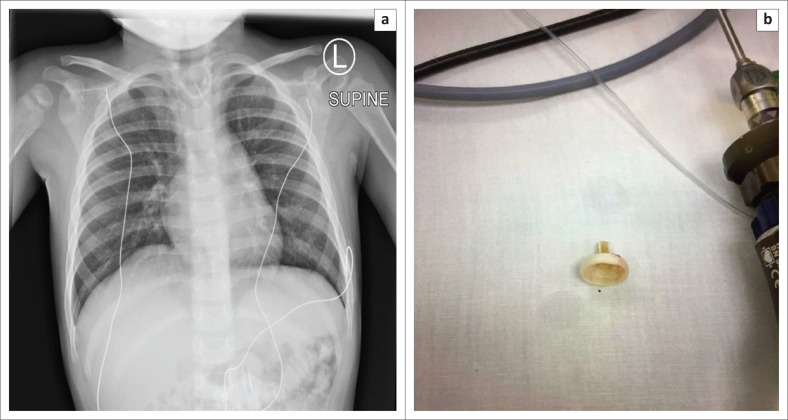
Chest radiograph (a) of a 4-year-old child showing a fractured and migrated gastrostomy tube (G-tube) component (b) in the cervical oesophagus. Note that the tracheal air shadow is seen separately, as the migrated G-tube component can mimic a tracheostomy tube.

Medical tubes and devices are intentionally placed in the aerodigestive tract (tracheostomy tube, feeding tube, stents, gastrostomy tube, etc.) for various support and life-saving purposes. However, very rarely these devices can become fractured or migrate to unintended areas. Such complications are very rarely reported in the paediatric population, and only limited literature exists on this subject. Most of the devices that have fractured and/or migrated are either tracheostomy tubes^44,45^ or oesophageal stents.^46^ To the best of our knowledge, we document the first case of a fractured and migrated gastrostomy tube component into the cervical oesophagus (Figure 18a and b). It is important for radiologists to recognise and immediately report such complications to prevent unwarranted complications.

## Conclusion

Radiography is the initial and most commonly used imaging modality for the evaluation of ingested or aspirated FBs in paediatric patients, with fluoroscopy and MDCT providing ancillary support in complex cases. It is essential for the radiologist to differentiate BB ingestion from coins, as the former requires an emergency diagnosis and removal to prevent life-threatening injury. It is prudent to remember that not all patients with FBA present with a straightforward history, and hence, prompt recognition of the secondary radiographic signs of FBA is essential to avoid unwarranted morbidity and mortality.
